# Environmentally Friendly g-C_3_N_4_/Sepiolite Fiber for Enhanced Degradation of Dye under Visible Light

**DOI:** 10.3390/molecules27082464

**Published:** 2022-04-11

**Authors:** Jiayue Sun, Lianying Wang, Simei Lu, Zhuoyuan Wang, Menglin Chen, Weixia Liang, Xiu Lin, Xiangfeng Lin

**Affiliations:** 1 Key Laboratory of Ecology of Rare and Endangered Species and Environmental Protection, Guangxi Normal University, Ministry of Education, Guilin 541004, China; davey0116@163.com (J.S.); wanglianying0217@163.com (L.W.); 9911006@126.com (S.L.); 2 School of Environment and Resource, Guangxi Normal University, Guilin 541004, China; 3 School of Chemistry and Pharmaceutical Science, Guangxi Normal University, Guilin 541004, China; hlx1975@163.com; 4 School of Medicine and Health, Guangxi Vocational & Technical Institute of Industry, Nanning 530001, China; 5 Guangxi Key Laboratory of Spatial Information and Geomatics, Guilin 541004, China; 6 Hospital, Guilin University of Technology, Guilin 541004, China; 7 Department of Public Health, International College, Krirk University, Bangkok 10220, Thailand

**Keywords:** g-C_3_N_4_, sepiolite fiber, dye, visible light, active species

## Abstract

Herein, novel visible light active graphitic carbon nitride (g-C_3_N_4_)/sepiolite fiber (CN/SS) composites were fabricated via a facile calcination route, exploiting melamine and thiourea as precursors, and sepiolite fiber as support, for efficient degradation of organic dye methylene blue (MB). The as-prepared CN/SS composites were characterized by various characterization techniques based on structural and microstructural analyses. The effects of CN loading amount, catalyst dosage and initial concentration of dye on the removal rate of dye under visible light were systematically studied. The removal rate of MB was as high as 99.5%, 99.6% and 99.6% over the composites when the CN loading amount, catalyst dosage and initial concentration of dye were 20% (mass percent), 0.1 g, and 15 mg/L in 120 min, respectively. The active species scavenging experiments and electron paramagnetic resonance (EPR) measurement indicated that the holes (h^+^), hydroxyl radical (·OH) and superoxide radicals (·O_2_^−^) were the main active species. This study provides for the design of low-cost, environmentally friendly and highly efficient catalysts for the removal of organic dye.

## 1. Introduction

Due to the rapid development of the textile industry and printing and dyeing industries, more and more effluents containing dyes and their intermediates are released into the environment. If not properly disposed, these effluents can be toxic to aquatic life, because even dye wastewater in low concentrations leads to an increase in water color and subsequent decrease in the penetration of sunlight, which will eventually interfere with photosynthesis [1,2]. Kiernan has confirmed that there are interactions between the dye molecules and cell membrane, and the former can penetrate the latter to enter into the cell interior and cause toxic reactions [3]. It will seriously threaten human health and destroy the balance of the ecosystem if the dye stays in the water bodies for a long time.

Various methods have been developed to eliminate dyes from textile wastewater; however, the advantages and disadvantages of wastewater treatments coexist [4]. For instance, adsorption has been proven to be an effective method for the removal of organic dyes from water because of its eco-friendliness and the low cost of conventional adsorbents [5]. New adsorbents with metal–organic frameworks (MOFs) have also been reported [6]. However, the dyes are not destroyed by adsorption [4,7]. The dye molecules can be destroyed in the advanced oxidation processes (AOPs), such as the photocatalytic process [4], in which dyes are broken down into CO_2_ and H_2_O or some other intermediate product. During the photocatalysis process, active and non-selective species such as the superoxide (·O_2_^−^) and hydroxyl radical (·OH) play the role of destroying organic pollutants [8,9].

The key to efficient dye degradation is the photocatalyst. Many types of photocatalysts have been proven effective in dye removal, among which semiconductors are the main type of catalysts [10]. Therefore, the design of the semiconductors has recently received considerable attention from researchers. The most commonly used semiconductors are titanium dioxide (TiO_2_); however, it cannot use visible light due to its relatively wide band gap energy (3.0–3.3 eV) and requires ultraviolet excitation. Various catalysts excited by visible light have been developed, including metal oxide or non-metal semiconductors, metal sulfide semiconductors, heterostructures, quantum dots, semiconductor oxide/quantum dot composites, magnetic semiconductor materials, and so on [11]. Among these semiconductors, recently, g-C_3_N_4_ is a topic of interest [12].

g-C_3_N_4_ has been widely applied for environmental remediation because of facile preparation, cost-effectiveness, unique energy-band structure [13,14], good electronic and chemical properties and high thermal stability. These characteristics make it a free metal catalysis for organic photodegradation [15]. g-C_3_N_4_ is the semiconductor with a narrow bandgap of 2.7 eV, which facilitates the absorbance of the visible light spectrum from solar light. Moreover, g-C_3_N_4_ exhibits nontoxic and environmentally friendly characteristics [16]. However, its low surface area and low quantum efficiency have limited its visible light utilization, and its poor charge separation and transfer efficiency severely restrict its degradation performance [13,17]. To overcome these disadvantages in photocatalysis, many strategies have been developed to improve its photocatalytic efficiency by means of metal/non-metal element doping, composites constructing, vacancy defects, functional groups introducing, morphology changing, etc. [18,19].

Conversely, and recently, employing natural mineral materials as the catalyst carrier has been extensively accepted as an efficient approach to settle the above-mentioned problems because of their widespread sources, low cost, superior adsorption ability, and excellent thermal and chemical stability. Sepiolite, as a clay mineral, with massive pores and large surface, due to its 2:1-type layered and fibrous structure, is composed of the acidic [SiO_4_] and alkalescent [MgO_6_], which shows superior adsorption capacity. Furthermore, its superior adsorption activity is beneficial to the photocatalytic degradation process [20,21]. Furthermore, the sepiolite is abundant, non-toxic [22]. The presence of the Si-OH covering on the sepiolite surface makes it a promising support for large quantities of metals and metal oxides [23,24,25,26], meanwhile, one great advantage of the sepiolite is that it is low cost, easy to obtain, and it is environmentally friendly [24,27,28,29]. It was found that the loading of g-C_3_N_4_ and noble metal on the surface of sepiolite can enhance its photocatalytic activity [26].

g-C_3_N_4_ catalysts are generally first fabricated by pyrolysis of melamine at 450 [30] or 550 °C [31,32,33] for 4 h, and then loaded onto TiO_2_ [30], magnetospheres/C [31], Fe/kaolinite [32], Fe_2_O_3_ [33] by mixing or annealing the g-C_3_N_4_ and supporter, and so on. Here, in our work, the g-C_3_N_4_/sepiolite was prepared by one-step calcination. In this work, we developed, as far as we know, for the first time, a non-metal semiconductor g-C_3_N_4_ loading on a sepiolite fiber by varying the composition of g-C_3_N_4_ to improve the efficient degradation of organic dye methylene blue under visible light. The sepiolite fiber here lowered the cost of the photocatalysts.

## 2. Materials and Methods

### 2.1. Materials

The sepiolite fiber was purchased from Neixiang Xinglei Sepiolte Co., Ltd., Nanyang, Henan Province, China. Melamine (AR, 99%), methylene blue (BS) and thiourea (AR, 99%) were purchased from Sinopharm Chemical Reagent Co., Ltd., Shanghai, China. Tert-butanol (AR, 99%) and disodium ethylene diamine tetraacetate (EDTA, AR, 99%) were purchased from Xilong Chemical Co., Ltd., Shantou, China. All the chemical reagents were used without further purification. Distilled water was used throughout the experiment.

### 2.2. Samples Preparation

#### 2.2.1. Pretreatment of SS

The raw SS was pretreated before use. Then, 20.0 g SS and 100 mL water were placed into conical flask and stirred for 24 h at room temperature, and then the upper suspended matters were discarded, the remaining deposits were filtered and washed three times by distilled water, and finally, the SS was dried in a drying oven for 24 h at 105 °C.

#### 2.2.2. g-C_3_N_4_/SS Synthesis

The typical preparation process of the g-C_3_N_4_/SS composites is as follows. At first, 2 g melamine, 3 g thiourea and 5 g pretreated SS powder, which were equivalent to 20 wt% of g-C_3_N_4_ in the composites (the pre-experiments showed that the yield of g-C_3_N_4_ was 20%, and the mass ratio of melamine and thiourea was 2:3), were well mixed and placed in 25 mL water, subsequently stirred and heated until the water evaporated completely. Then, the mixture was grinded and placed into a quartz boat and transferred into the muffle furnace and heated at 600 °C in nitrogen atmosphere for 3 h with a heating rate of 5 °C/min. Finally, the 20% CN/SS composites were obtained after cooling down, and they were milled. Other CN/SS composites with different CN loading were prepared using the same procedure, only changing the mass of melamine and thiourea.

### 2.3. Characterization

A D8 Bruker X-ray diffractometer (Bruker Corporation, Billerica, MA, USA) was used to check the structure and crystallinity of the raw SS and synthesized CN/SS. Fourier transform infrared (FTIR) spectrometer (Spectrum Two, Perkin-Elmer Corporation, Waltham, MA, USA) was utilized to identify functional groups attached to the surface of the composite. The X-ray photoelectron spectroscopy (XPS) measurements were obtained by a PHI 5000C ESCA System X-ray photoelectron spectroscopy (Phisical Electronic Corporation, Tumwater, WA, USA). UV–Vis diffuse-reflectance spectroscopy experiments were performed on a Hitachi U4100 UV spectrophotometer (Hitachi Ltd., Tokyo, Japan) equipped with an integrating sphere. The Brunauer Emmett Teller (BET) surface areas analyses were carried out by N_2_ adsorption–desorption using an automated gas sorption analyzer (QuadraSorb SI, Quantachrome Corporation, Boynton Beach, FL, USA). The morphology of the catalyst samples was revealed by a FEI Titan 80-300 transmission electron microscope (FEI Corporation, Hillsboro, FL, USA). Electron paramagnetic resonance (EPR) spectra was recorded on a JEOL JES FA200 EPR Spectrometer (JEOL Ltd., Akishima, Japan). The concentration of dye was measured on a TU-1901 UV-Vis spectrophotometer (Beijing Purkinje General Instrument Co., Ltd., Beijing, China).

### 2.4. Photocatalytic Degradation of Methylene Blue

The photocatalysis tests were carried out in a multi-position photochemical reaction system (Zhongjiao Jinyuan Technology Co., Ltd., Beijing, China) equipped with a 300 W xenon lamp (420 nm < *λ* < 760 nm, with the 420 nm cutoff filter) and water cooling unit. A proper mass of (0.05, 0.1, 0.15 g) CN/SS was added into a quartz reactor containing 50 mL methylene blue (the concentration was changed at different reaction conditions: 15, 30, 50 and 80 mg/L), and the suspension was stirred in the dark for 60 min to reach the adsorption–desorption equilibrium. Then, the light source was turned on, the suspension was sampled for 3 mL in a given time interval, and then it was filtered with a microporous membrane with a 0.22 µm pore diameter. The absorbance of methylene blue was measured by the TU-1901 UV-Vis spectrophotometer; subsequently, the removal rate of dye was calculated.

### 2.5. Trapping Experiments of Active Species

Active species trapping experiments were carried out to identify the free radical in the photocatalytic process. Tert-butanol (t-But) and EDTA-2Na were utilized as active species capture agents for the hydrocarbyl radical (·OH) and hole (h^+^), and both of the concentrations of t-But and EDTA-2Na were 0.1 mol/L. The EPR measurement was performed to confirm the superoxide radicals ·O_2_^−^) and ·OH.

## 3. Results and Discussion

### 3.1. Structure and Morphology Analysis

**Figure 1:**
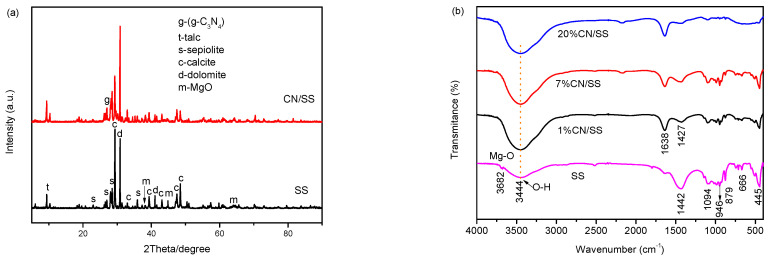
XRD pattern (SS and 20% CN/SS) (**a**) and FTIR spectra for SS, 1% CN/SS, 7% CN/SS and 20% CN/SS composites (**b**).

The XRD patterns of the samples are shown in Figure 1a. The peaks present at 2θ with values of 22.8°, 26.4°, 28.5°and 35.8° can be indexed as the (330), (080), (331), (022) planes of sepiolite [34]. The diffraction peak at 2θ value of 9.4° is attributed to the talc [35]. The reflections at 2θ = 38.3°, 44.8° and 64.4° match the characteristic peaks of MgO [36]. The peaks at 2θ = 29.3°, 33.1°, 39.4°, 43.1°, 47.3° and 48.4° possibly belong to the characteristics peak of calcite [37,38,39,40], while the peaks at 2θ = 30.8°, 41° and 50.4° are dolomite impurities [39,40]. After modification, the diffraction peak at 2θ = 27.1° is ascribed to the (002) planes of g-C3N4 [32]; however, the diffraction intensity of g-C_3_N_4_ is low because the intensity of the calcite and dolomite in the sepiolite fiber is too strong. The intensity of some diffraction peaks in SS changes may be due to the changes in the structure of the sepiolite fiber after calcination at 600 °C.

The functional groups in samples were further analyzed by FTIR measurement. The FTIR spectra of SS, 1% CN/SS, 7% CN/SS and 20% CN/SS composites are presented in Figure 1b. The absorption peaks at 3682 and 666 cm^−1^ correspond to the vibrations of Mg-OH groups stemmed from the Mg-O octahedron (tri-(Mg_3_OH)) [41,42,43]. The band around 3444 cm^−1^ is ascribed to the vibration of the O-H group, which originates from the interbedded water [44]. The band at 1440 cm^−^^1^ belongs to the vibration of CO_3_ derived from the carbonate impurities [40]. The bands at 1094 and 946 cm^−1^ are associated with the stretching vibration of the Si-O band [45,46]. The peak at 879 cm^−1^ is attributed to the Ca-O [36], which is consistent with the existence of the calcite impurity derived from XRD analysis (Figure 1a). The peak at 445 cm^−1^ comes from the deformation mode of MgO_6_ octahedral units previously reported by Walczyk [40]. The peaks at 1638 and 1427 cm^−1^ are assigned to the C=N stretching vibration [18].

**Figure 2:**
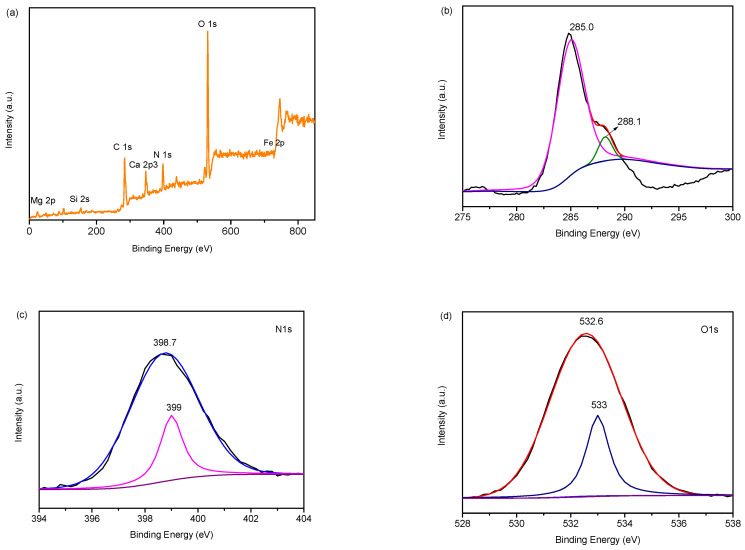
XPS spectra (**a**) and high-resolution of C1s (**b**), N1s (**c**), and O1s (**d**).

The composition and chemical valence of the 20% CN/SS composite was measured by XPS, and the results are presented in Figure 2a–d. The characteristic peaks of C1s, N1s, O1s, Fe2p, Si2s, Ca2p3 and Mg2p can be observed in Figure 2a, indicating that the g-C_3_N_4_ has been successfully loaded onto the sepiolite. The high resolution of the C1s spectra (Figure 2b) can be deconvoluted into two different peaks at 288.1 and 285 eV. The peak at 288.1 eV is ascribed to the sp2 hybrid orbital (N-C=N) [22], while the peak at 285 eV belongs to the surface-attached carbon (sp3 C-C) [47]. The N1s spectra (Figure 2c) can also be deconvoluted into two different peaks at 399 and 398.7 eV; they are associated with the N atom from the N-(C_3_) and the C=N-C groups, respectively [22,48,49]. The two peaks at 532.6 and 533 eV deconvoluted from the O1s spectra (Figure 2d) correspond to the O-H and Si-O-Si bonds, respectively, which are derived from the adsorbed water on the surface and clay, respectively [49,50].

**Figure 3:**
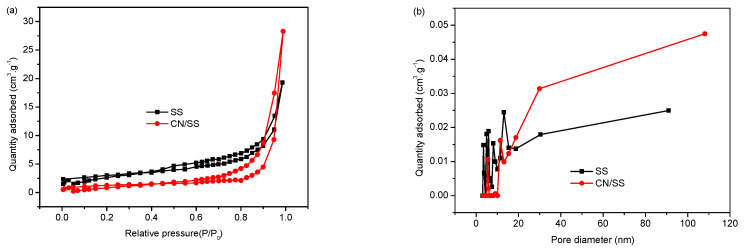
BJH nitrogen adsorption–desorption isotherms (**a**) and the corresponding pore size distribution plots (**b**).

The nitrogen adsorption–desorption test was carried out to illustrate the surface area and pore structure of the materials. The specific surface area of SS is 10.23 m^2^/g, after loading by CN, the specific surface area of 20% CN/SS decreases to 4.36 m^2^/g, suggesting that the CN enters into the pores of SS and then blocks the access of nitrogen molecules to the pores and channels of SS (see Figure 3a). BJH nitrogen adsorption–desorption curves of SS shows that the material has an obvious hysteresis loop between P/P_0_ = 0–0.2 and P/P_0_ = 0.45–1, belonging to the type IV H3 hysteresis ring, showing the typical characteristics of non-rigid aggregates, that is, belonging to the typical mesoporous materials. It can also be seen from the pore size distribution diagram (Figure 3b) that the pore size is in the range from 3 to 90.9 nm and mainly concentrates between 3–15.3 nm, indicating that the SS is a mesoporous material. For the sample 20% CN/SS, its BJH nitrogen adsorption–desorption isotherm (Figure 3a) corresponds to a type IV H4 hysteresis loop, demonstrating the narrow slit-like pores. The pore size distribution of 20% CN/SS is between 3.4–108 nm and especially concentrates between 3.4–10 nm (Figure 3b), indicating that further loading with CN leads to more small-sized mesopores. This signifies that the specific surface area may not be the main factor affecting the activity of the catalyst [47].

**Figure 4:**
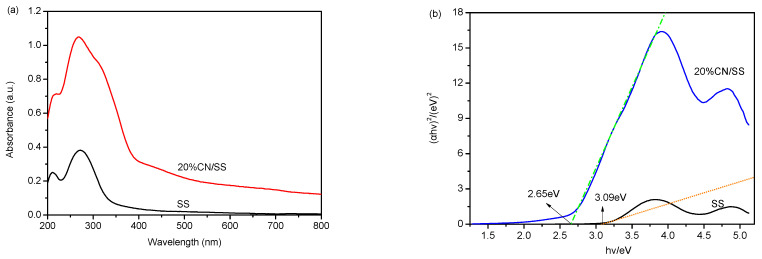
Ultraviolet–visible diffuse reflectance of SS and 20% CN/SS (**a**); the plot of (*αhν*)^2^ versus *hν* (**b**).

The light absorption properties of a photocatalyst are crucial in its photocatalytic activity since incident photons absorbed by the photocatalyst will determine the excitation, transfer, and redox abilities of the charge carriers [51]. Figure 4a shows the UV–Vis DRS of SS and 20% CN/SS. The light absorption of sepiolite is almost in the UV region; only slight absorption within the visible light region is observed. For the 20% CN/SS sample, after modification by g-C_3_N_4_, it extends the absorption band from UV to the visible light region. The increased light absorption is due to the charge transfer from the N2p orbit to C2p orbit [51,52], also suggesting the formation of intermolecular interactions between g-C_3_N_4_ and the sepiolite fiber. The band gap energies of SS and 20% CN/SS are evaluated by the Kubelka–Munk function [14] using the following formula (1):

*αhν* = *A* (*hν* − *E_g_*)^*n*/2^ (1)

where *α* is the absorption coefficient, *h* is the Planck’ constant, *ν* is the light frequency, *A* is constant and *E_g_* is the band gap energy. Furthermore, the value *n* is dependent on the type of optical transition of the semiconductor (*n* = 1 for direct transition and *n* = 4 for indirect transition) [26,53]. The band gap energy (*E_g_*) of SS and 20% CN/SS are 3.09 eV and 2.65 eV, respectively, which are derived from a plot of (*αhν*)^2^ versus *hν* (Figure 4b). The result of the UV–Vis DRS measurement shows that the 20% CN/SS sample enhances the optical absorption performance in the visible light region, and it is foreseeable that the 20% CN/SS will improve the utilization efficiency of the simulated sunlight.

**Figure 5:**
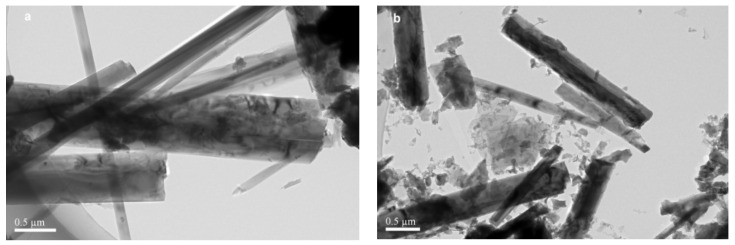
TEM image of SS (**a**) and 20% CN/SS (**b**).

The surface morphology of the raw SS and 20% CN/SS was investigated by TEM. The raw SS shows a one-dimensional belt-shaped microstructure with a diameter varying between 136 and 818 nm (Figure 5a). After modification by g-C_3_N_4_, the dimensional structure of raw SS is broken, it becomes looser than that of the raw SS, and the surface of 20% CN/SS is rough (Figure 5b). This indicates that the morphology of the raw SS is changed by loading of g-C_3_N_4_ and calcination.

### 3.2. Photocatalytic Degradation

#### 3.2.1. Effect of CN Loading

**Figure 6:**
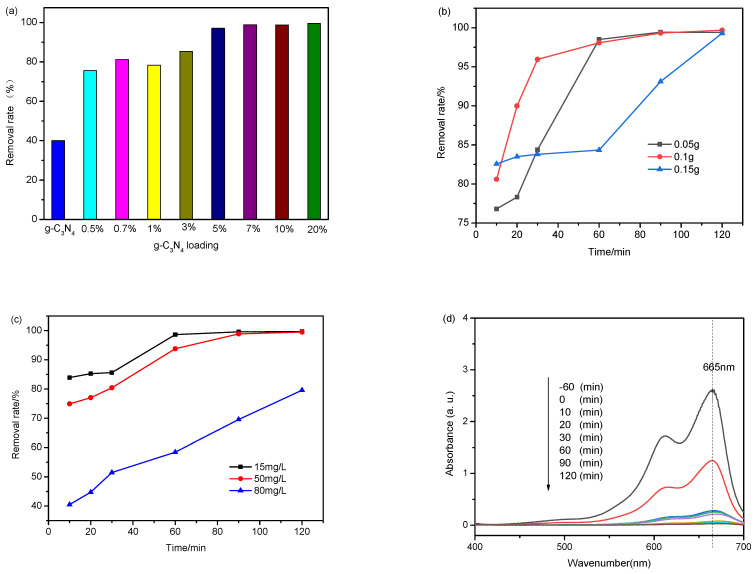
Effect of g-C_3_N_4_ loading (**a**), catalyst dosage (**b**) and initial concentration (**c**) in photocatalytic degradation of MB on 20% CN/SS, UV–Vis spectra of the residual dyes (0.05 g 20% CN/SS, initial dye concentration: 30 mg/L) (**d**).

The effect of CN loading on the dye removal rate is illustrated in Figure 6a at 0.05 g catalysts and 30 mg/L initial concentration. The removal rate of dye is increased with the increase in CN loading from 0.5% to 20%, except the 1% loading is slightly lower. However, the removal rates are almost the same in the range from 97.1% to 99.5% when the CN loading is increased from 5% to 20%. The 20% CN/SS sample shows the highest catalytic activity. Therefore, the 20% CN/SS sample was used in other photocatalytic reactions. The pure CN shows lower photocatalytic activity, indicating that the combinations of CN and SS promotes charge separation, which is beneficial for the removal efficiency improvement.

#### 3.2.2. Effect of Catalyst Dosage

The effect of varied dosages of 20% CN/SS on MB degradation was investigated. Varying amounts of 20% CN/SS were placed into the 50 mL (30 mg/L) MB solution. According to the results exhibited in Figure 6b, the highest removal rate is observed in the 20% CN/SS dose of 0.15 g in the first 10 min. However, the highest removal rates of 90% and 95.9% were achieved in 20 and 30 min for the dose of 0.1 g. After 120 min of reaction, the removal rate of dye by different doses of catalysts are 99.4% (0.05 g), 99.6% (0.1 g) and 99.3% (0.15 g). In the first 10 min, increasing the amount of catalyst dose leads to the increase in dye removal efficiency under visible light, ascribing to the increase in the available surface area and the number of active sites on the catalyst surface (to adsorb optical photons). Therefore, more holes (h^+^) are produced by visible light irradiation, which leads to an increase in other reactive species such as ·O_2_^−^ and ·OH and a consequent increase in removal rate. However, increasing the amount of catalyst dose reduces the water transparency, hindering the incidence of light and adsorption of optical photons, which consequently results in decreasing the removal rate. Hence, the removal rate for a catalyst dose of 0.15 g is lower than that of 0.05 and 0.1 g at the reaction time of 30–90 min. After irradiation for 120 min, the removal rate from different doses of catalysts are almost the same due to the extremely low dye concentration left in the solution.

#### 3.2.3. Effect of Initial Dye Concentration

In industrial effluents, the concentration of dyes is an important operating parameter. For this reason, it is interesting to investigate the effect of initial dye concentration on the performance of the catalyst. Therefore, different concentrations of 15, 50 and 80 mg/L of MB dye solution were prepared and degraded under visible light on a catalyst dose of 0.05 g; the catalyst used here was 20% CN/SS, and the results are presented in Figure 6c. As can be seen, upon irradiation for 10 min, the removal rate in 15, 50 and 80 mg/L of MB was obtained at 83.9%, 74.9% and 40.5%, respectively. By increasing the concentration, the photocatalytic performance of the catalyst decreased because the surface of the photocatalyst was saturated with dye molecules and the water transparency was reduced [54]. However, as the reaction proceeded, the dye concentration in the solution decreased, which led to the increase in the water transparency and removal rate.

#### 3.2.4. UV–Vis Spectra of the Residual Dyes

The UV–Vis spectra of the residual dyes under different irradiation time is shown in Figure 6d. The surface of the 20% CN/SS sample reaches adsorption equilibrium with MB molecules in dark conditions after 60 min. After 60 min of visible light irradiation, the absorbance of the dye significantly decreases. It nearly disappears at the reaction time of 120 min.

### 3.3. Reusability of CN/SS

**Figure 7:**
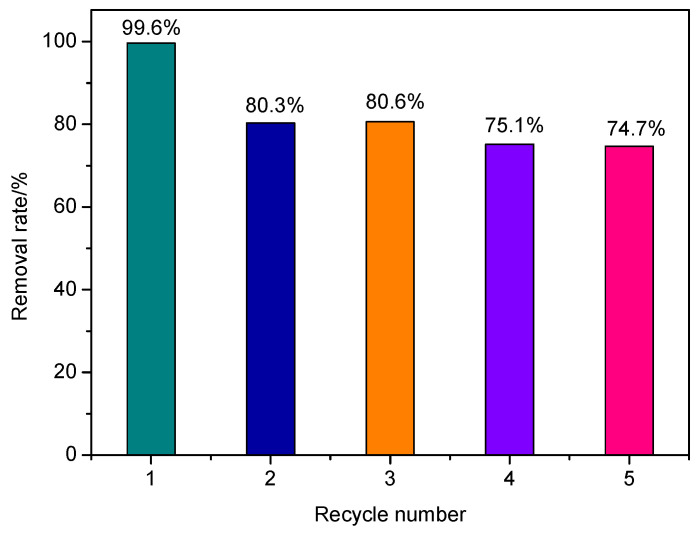
Reusability studies for methylene blue degradation over 20% CN/SS under visible light.

The reusability of the photocatalyst is important in its practical application. Thus, the cyclic photo degradation dye-reutilization performance of 20% CN/SS was tested, and the results are presented in Figure 7. The second and third cycle shows a removal rate of 80.3% and 80.6%, respectively. After five successive cycles for methylene blue degradation, the as-prepared 20% CN/SS remains at 74.7% of its activity. Its activity decreases by 25% compared to the first use. However, on the whole, it achieves a relatively satisfactory reusability of the photocatalyst.

### 3.4. Investigation of Reactive Species and Photocatalytic Degradation Mechanism

**Figure 8:**
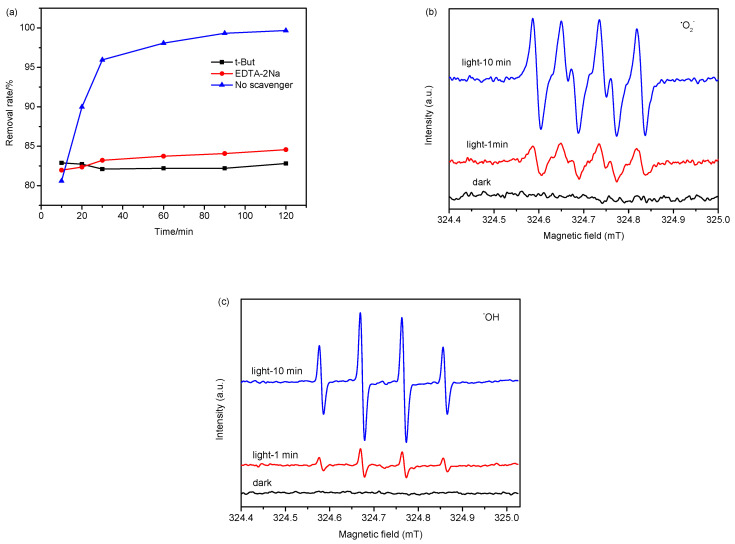
The trapping experiments of reactive species by t-But and EDTA-2Na (**a**), (0.1 g 20% CN/SS, dye: 30 mg/L, 50 mL), EPR spectra of 20% CN/SS in ·O_2_^−^-DMPO in methanol (**b**), and ·OH-DMPO in water (**c**). (DMPO = 100 µL/mL both in **b** and **c**).

To investigate the photocatalytic degradation mechanism, reactive species scavenger experiments were carried out. EDTA-2Na and t-But were used as the captures of h^+^ and ·OH, respectively. Figure 8a presents the removal rate of methylene blue on 20% CN/SS after the addition of different scavengers. With the introduction of EDTA-2Na as the h^+^ scavenger, the dye photodegradation was suppressed. With the addition of t-But as the ·OH scavenger, the photocatalytic activity of CN/SS decreased, indicating that h^+^ and ·OH are involved in the dye degradation process.

Furthermore, the active electrons in the conduction band (CB) can react with dissolved oxygen molecules to generate the ·O_2_^−^ radical [55]. Therefore, the EPR measurements were conducted to analyze the produced ·O_2_^−^ active species, using 5,5-dimethyl-1-pyrroline N-oxide (DMPO) as the radical scavenger. Figure 8b displays the DMPO-·O_2_^−^ adduct signals of 20% CN/SS in dark and light irradiation for 1 and 10 min, respectively. The signals of DMPO-·O_2_^−^ are not observed in the dark; however, the obvious signals are obtained after 1 min of visible light irradiation, and then the signals are remarkably enhanced at the irradiation time of 10 min, indicating that the generation of ·O_2_^−^ is related to the light irradiation time to some extent. The ·OH radical is also confirmed by EPR test (Figure 8c). For the signals of DMPO-·OH, similarly, there are no recognizable peaks appeared in dark. Then, they appear more and more stronger with the increase in irradiation time. The EPR experiments confirm that a mass of reactive species with strong oxidizing ability is involved in dye degradation under visible light irradiation. It is proven by the scavenger and EPR experiments that h^+^, ·O_2_^−^ and ·OH radicals are all main oxidative species in the photocatalytic dye process.

**Figure 9:**
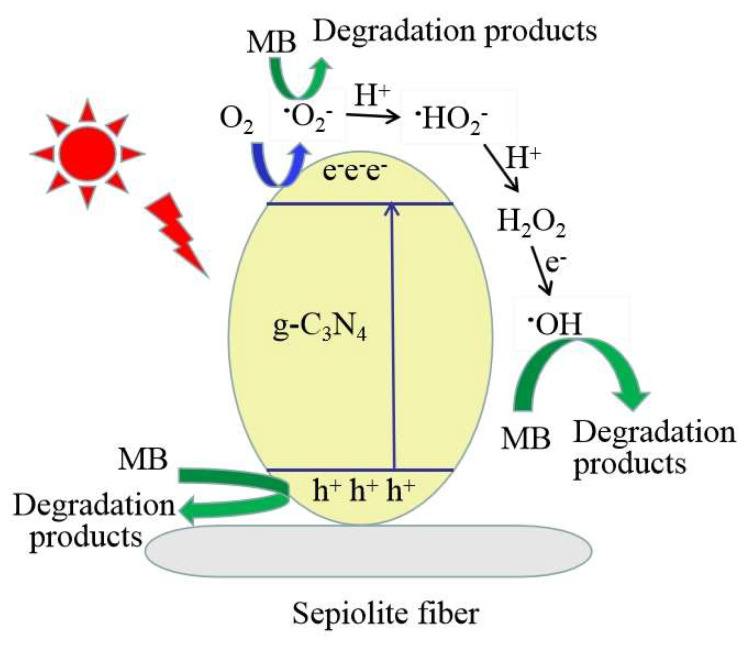
Schematic diagram of the photocatalytic degradation mechanism.

Based on the above experiments, the possible photocatalytic degradation mechanism of methylene blue over 20% CN/SS is proposed (Figure 9). Under visible light irradiation, the valence band (VB) electrons (e^−^) of g-C_3_N_4_ in the photocatalyst is easily excited to the conduction band (CB) by photons, leaving the h^+^ in the VB. The h^+^ directly oxidized the pollutants. Moreover, the new electron traps generated between the sepiolite and g-C_3_N_4_ might accept electrons to avoid the recombination of photogenerated electron-hole pairs of g-C_3_N_4_ [26]; therefore, the holes could effectively degrade the pollutants. The electrons can reduce the dissolved O_2_ in water to form ·O_2_^−^, and meanwhile, the ·O_2_^−^ reactive species can react with H^+^ or H_2_O to produce ·OH radicals. Conversely, the surface of the sepiolite fibers are negatively charged [56], there is electrostatic repulsion between the negatively charged electrons and negatively charged surface of SS, and electrostatic attraction exists between the positively charged holes and the negatively charged surface of SS. Hence, the photogenerated electron-hole pairs of g-C_3_N_4_ can migrate efficiently, which is in agreement with the kaolinite/g-C_3_N_4_ composite [57].

Based on the above analysis, the possible photocatalytic reaction processes are proposed, as shown in Equations (2)–(7).

CN/SS + visible light→e^−^ + h^+^ (2)

O_2_ + e^−^→·O_2_^−^ (3)

·O_2_^−^ + H^+^→·HO_2_ (4)

·HO_2_ + H^+^→H_2_O_2_ (5)

H_2_O_2_ + e^−^→·OH + OH^+^ (6)

·O_2_^−^/h^+^/·OH + pollutants→degraded products (7)

## 4. Conclusions

In this study, a low-cost and efficient photocatalyst CN/SS was fabricated and used for the removal of organic dye MB from an aqueous solution. The photocatalyst was characterized by various analytical methods such as XRD, FTIR, XPS, BET, TEM and UV–Vis. Photocatalytic activity experiments showed that the removal rate of MB was influenced by CN loading amount, catalyst dosage and initial dye concentration under visible light, and the removal rates were in the range of 74.9% to 99.6%. The active species (h^+^, ·OH and ·O_2_^−^) were tested by scavenging and EPR experiments. The CN/SS displayed a relatively satisfactory reusability, which is a key parameter for practical application. This photocatalyst is environmentally friendly, low cost and highly efficient, and it would be promising in the degradation of organic dye under sunlight.

## Data Availability

The data can be made available upon reasonable request.

## Sample Availability

Samples of the compounds are not available from the authors.
